# Modeling lung endothelial dysfunction in sepsis‐associated ARDS using a microphysiological system

**DOI:** 10.14814/phy2.16134

**Published:** 2024-07-09

**Authors:** Nai‐Wen Liang, Carole Wilson, Brooke Davis, Ian Wolf, Tonela Qyli, Joy Moy, David J. Beebe, Lynn M. Schnapp, Sheena C. Kerr, Hilary E. Faust

**Affiliations:** ^1^ Department of Biomedical Engineering University of Wisconsin Madison Wisconsin USA; ^2^ Division of Allergy, Pulmonary and Critical Care, Department of Medicine, School of Medicine and Public Health University of Wisconsin Madison Wisconsin USA; ^3^ College of Letters and Science University of Wisconsin Madison Wisconsin USA; ^4^ Department of anesthesiology and Perioperative Care University of California Irvine Irvine USA; ^5^ Department of Medicine, School of Medicine and Public Health University of Wisconsin Madison Wisconsin USA; ^6^ Department of Pathology and Laboratory Medicine University of Wisconsin Madison Wisconsin USA; ^7^ Carbone Cancer Center University of Wisconsin Madison Wisconsin USA

**Keywords:** ARDS, endothelial dysfunction, microphysiological system, sepsis

## Abstract

Endothelial dysfunction is a critical feature of acute respiratory distress syndrome (ARDS) associated with higher disease severity and worse outcomes. Preclinical in vivo models of sepsis and ARDS have failed to yield useful therapies in humans, perhaps due to interspecies differences in inflammatory responses and heterogeneity of human host responses. Use of microphysiological systems (MPS) to investigate lung endothelial function may shed light on underlying mechanisms and targeted treatments for ARDS. We assessed the response to plasma from critically ill sepsis patients in our lung endothelial MPS through measurement of endothelial permeability, expression of adhesion molecules, and inflammatory cytokine secretion. Sepsis plasma induced areas of endothelial cell (EC) contraction, loss of cellular coverage, and luminal defects. EC barrier function was significantly worse following incubation with sepsis plasma compared to healthy plasma. EC ICAM‐1 expression, IL‐6 and soluble ICAM‐1 secretion increased significantly more after incubation with sepsis plasma compared with healthy plasma. Plasma from sepsis patients who developed ARDS further increased IL‐6 and sICAM‐1 compared to plasma from sepsis patients without ARDS and healthy plasma. Our results demonstrate the proof of concept that lung endothelial MPS can enable interrogation of specific mechanisms of endothelial dysfunction that promote ARDS in sepsis patients.

## 1 INTRODUCTION

Sepsis is the leading cause of acute respiratory distress syndrome (ARDS), a syndrome resulting in up to 45% mortality without any available targeted therapies (Bellani et al., 2016; Ranieri et al., 2012). Endothelial dysfunction is an important component of both ARDS and sepsis and confers worse disease severity and higher mortality. Markers of endothelial dysfunction are associated with the hyperinflammatory subphenotype of ARDS (Bos et al., 2017; Calfee et al., 2014), which exhibits significantly higher mortality. Endothelial dysfunction may directly cause worse outcomes in diverse inflammatory conditions including sepsis, the leading risk factor for ARDS (Wu et al., 2021). Given its critical role in the development of ARDS in sepsis patients, endothelial dysfunction has been proposed as a treatable trait that may hold the potential to improve outcomes in sepsis and sepsis‐associated ARDS if effectively targeted (Bos et al., 2022).

Understanding and targeting endothelial dysfunction in sepsis and ARDS is constrained by dual challenges: limited fidelity of animal and two‐dimensional culture models to recapitulate sepsis and ARDS; and the inaccessibility of human endothelial tissue to better define causal mechanisms in critically ill patients. Preclinical in vivo models of sepsis and ARDS have failed to yield useful targeted therapies in humans. Potential explanations for this include interspecies differences in inflammatory responses and heterogeneity of human host responses to infection (Soroush et al., 2020). Two‐dimensional in vitro models fail to convincingly mimic lung vasculature and heterogeneous host responses, factors with critical impacts on biological behavior and response to targeted therapies in other diseases (Palakshappa & Meyer, 2015). Biopsy of lung tissue and endothelium is rarely performed in fragile, critically ill patients, limiting insight into in vivo mechanisms of endothelial dysfunction (Jiménez‐Torres et al., 2016). New approaches are needed to identify the presence and underlying mechanisms of endothelial activation in sepsis in order to identify biomarkers and potential therapeutic targets.

In order to overcome these challenges, we combined a three‐dimensional lung endothelial microphysiological system (MPS) that recapitulates lung vascular architecture with plasma from sepsis patients to provide clinically relevant insight into sepsis‐induced lung endothelial dysfunction. The LumeNEXT platform used here consists of molded luminal structures in a collagen‐fibronectin hydrogel (Jiménez‐Torres et al., 2016). Seeding these luminal structures with primary human lung endothelial cells (ECs) results in creation of an endothelial microvessel that more accurately reproduces the critical tissue thought to mediate lung vascular injury in sepsis. Further, the microscale nature of the platform is well‐suited for working with small sample volumes enabling the analysis of limited patient samples. Culture of ECs in a can have a profound impact on cellular function (Bischel et al., 2014). Compared with 2‐dimensional traditional culture, ECs' culture in physiologically relevant three‐dimensional geometry generates significant differences in secretion of IL‐8, endothelin‐1, VEGF‐C, and other cytokines that reflect endothelial dysfunction, even in the absence of immune cells or flow (Bischel et al., 2014). LumeNEXT has been used to investigate bacterial (Hind et al., 2018), fungal (Hind et al., 2021), and parasitic infections (Humayun et al., 2022), demonstrating robust responses to infection and facilitating investigation into human responses to these organisms. However, this is the first time the system has been used to investigate endothelial responses to patient plasma samples in sepsis. Here we report use of a lung endothelial MPS to evaluate lung microvascular endothelial responses to plasma from sepsis patients (Figure S1) (Barkal et al., 2017). This allowed us to account for heterogeneity of human sepsis, including diverse infectious etiologies, sites of infection, severity of illness, and host responses. We hypothesized that plasma from critically ill sepsis patients would induce increased endothelial barrier dysfunction, inflammatory cytokine production, and vascular activation markers compared to plasma from healthy donors (“healthy plasma”).

## 2 METHODS

### 2.1 Study population

The study was approved by the University of Wisconsin (UW) Institutional Review Board (IRB 2021‐0974). We obtained plasma from ambulatory healthy subjects (“healthy plasma”) and from sepsis patients enrolled in the UW Sepsis Biobank (“sepsis plasma”), utilizing residual clinical lab specimens from the sample most proximal to ICU admission. The UW Sepsis Biobank enrolls critically ill patients who exhibit end‐organ dysfunction, as defined by dysfunction of two or more organs, with a strong suspicion of infection, indicated by clinical orders for cultures and antimicrobial treatments, according to the 2016 Surviving Sepsis Campaign guidelines (Rhodes et al., 2017). Written informed consent was obtained from patients or their surrogates within 96 h of ICU admission. Plasma was obtained from the UW Blood Bank from healthy blood donors under an existing protocol. Mortality was defined at 30 days after admission, and ARDS was defined within 5 days of ICU admission using the Berlin Definition (Ranieri et al., 2012). Patients with sepsis and ARDS are referred to as “Sepsis+ARDS,” while those without ARDS are termed “Sepsis without ARDS.”

### 2.2 Experimental procedures

Lumen construction: LumeNEXT devices were fabricated as previously described (Figure S1) (Barkal et al., 2017; Jiménez‐Torres et al., 2016) and filled with a collagen/fibronectin hydrogel to mold three‐dimensional lumens with diameter of 500 μm. The lumens were seeded with lung microvascular ECs (CC‐2527, Lonza, Basel, Switzerland), which are more clinically relevant to sepsis‐associated ARDS than other commonly used ECs such as human umbilical vein ECs. Devices were cultured in endothelial growth medium (EGM)‐2 MV culture medium (CC‐3202, Lonza) until confluence, resulting in a three‐dimensional endothelial lumen with circumferential cell coverage (Figure S1C, Videos S1 and S2).

### 2.3 Plasma processing, incubation, and assessment

Plasma from residual clinical lab specimens was collected and frozen at −80°C until the experiments were conducted. Thawed plasma was diluted 1:5 and then 1.6 μg/mL heparin was added to prevent coagulation. Plasma was diluted to prevent coagulation, with serial dilutions revealing 1:5 to be the least dilute concentration compatible with incubation in the lumens. Diluted plasma was incubated in the endothelial lumens for 16 h and then collected for further analysis. We quantified cytokine and endothelial activation markers relevant to inflammation, immune response, and endothelial activation that have previously been associated with sepsis‐associated ARDS in plasma before and after incubation in the MPS lumens using a custom multiplexed bead‐based ELISA (PPX‐10‐MX323RP, Luminex, Thermo Fisher). We defined “cytokine/inflammatory marker secretion” as the value obtained from the plasma after incubation in the endothelial MPS minus the value obtained from the plasma prior to incubation in MPS.

### 2.4 Endothelial lumen morphologic and barrier function assessment

**FIGURE 1:**
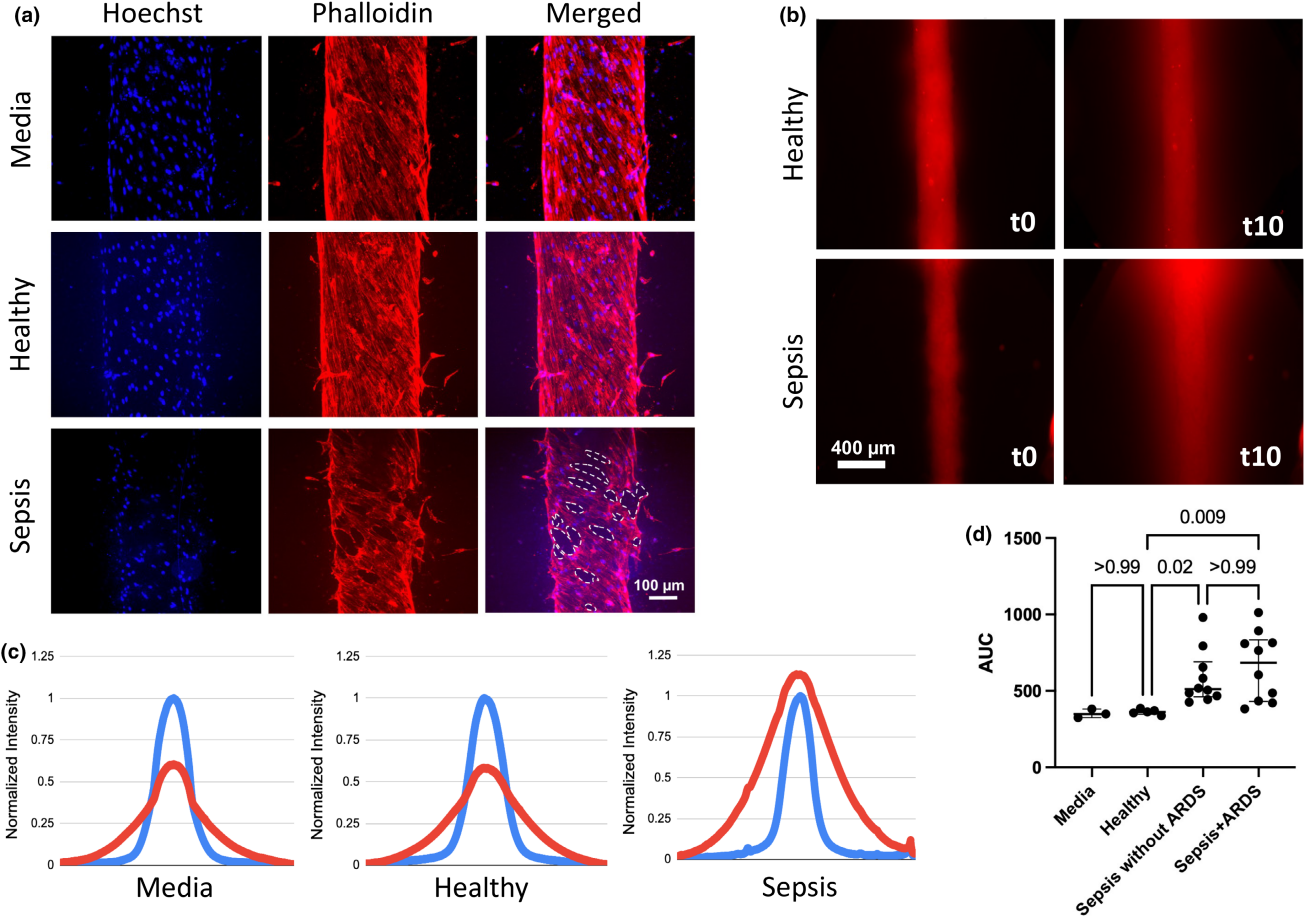
Lung endothelial MPS incubated with cell culture media (“media”), healthy donor plasma (“healthy”), and sepsis patient plasma (“sepsis”) for 16 h. (a) Staining of lumens incubated with media, healthy and sepsis plasma. Dashed white lines indicate holes in EC coverage in sepsis lumen. (b) Fluorescent dextran imaged just after addition to lumens (“t0”) and 10 min later (“t10”). (c) Plots of fluorescence intensity across lumens incubated with media, healthy and sepsis plasma. Intensity normalized to minimum (0) and maximum (1) at t0 (blue line) and t10 (red line). (d) Increased vascular permeability in lumens incubated with sepsis + ARDS and sepsis without ARDS plasma (*n* = 10 Sepsis+ARDS plasma, median 684.9, IQR 365.7; *n* = 10 Sepsis without ARDS plasma, median 511.9, IQR 164.6) compared with healthy plasma (*n* = 5 healthy controls, median 363.1, IQR 14.9, *p* = 0.009 for difference with Sepsis+ARDS plasma, *p* = 0.02 for difference with Sepsis without ARDS plasma) or media (median 349.5 IQR 27.9.)

EC barrier function was assessed by measuring diffusion of Texas Red‐labeled 50 kd dextran (Thermo Fisher Scientific, D1864, Waltham, MA) from the vascular lumen. Permeability was quantified as the area under the curve (AUC) of the fluorescence intensity plot at 10 min of dextran incubation normalized to the minimum (0) and maximum (1) of fluorescence at time 0 (Figure 1). Note that fluorescent dextran can be drawn from reservoir (devices ports) that are not captured in the images/plots, resulting in normalized intensity exceeding 1. The cause of this excess dextran is speculated to be a difference in osmotic pressure after excess leakage, but is out of the scope of this study and not discussed in detail here. To assess lumen integrity and cellular confluence, lumens were stained with nuclear (Hoechst, H1399, Thermo Fisher Scientific, Waltham, MA) and F‐actin dyes (Texas‐red phalloidin, T7471, Thermo Fisher Scientific, Waltham, MA), and imaged using a Nikon TI Eclipse microscope. Lumens were also stained with intracellular adhesion molecule 1 (ICAM‐1) (BBA3, R&D Systems, Minneapolis, MN) to determine the effect of sepsis plasma on vascular adhesion markers.

### 2.5 Statistical analysis

Differences in endothelial lumen permeability and ICAM expression were compared using nonparametric tests due to non‐normal distribution of results. Permeability was analyzed using Kruskal–Wallis test with multiple comparisons, while ICAM expression was analyzed with Mann–Whitney *U* test. Cytokine and inflammatory marker secretion levels were assessed using statistical tests appropriate for the data distribution. Specifically, the Student *t*‐test with unequal variance was utilized for comparing concentrations between healthy individuals and those with sepsis, assuming a normal distribution of results. The Kruskal–Wallis test was employed for comparing concentrations among healthy individuals, individuals with sepsis without ARDS, and individuals with sepsis+ARDS, considering non‐normally distributed results. All statistical tests were performed with GraphPad Prism (version 10.0.0 for Windows, GraphPad Software, Boston, Massachusetts USA, www.graphpad.com) using *α* = 0.05. Normality was assessed with QQ plots.

## 3 RESULTS

### 3.1 Patient characteristics

Twenty sepsis patients and five healthy donors were included in the study. Ten patients (50%) subsequently developed ARDS (sepsis+ARDS), and 6 (30%) died within 90 days. Source of infection was pulmonary in eight patients (40%) and extrapulmonary in 12 patients (60%). Eight patients (40%) developed shock prior to ICU admission. Other patient characteristics are summarized in Table S1.

### 3.2 Endothelial lumen morphology and permeability

Incubation of sepsis plasma induced marked cellular changes in ECs compared with incubation of healthy donor plasma (Figure 1a). ECs treated with sepsis plasma exhibited contraction in diameter, loss of cellular coverage, and luminal defects as demonstrated by F‐actin staining and lumen coverage analysis, compared with those incubated with healthy plasma. These findings correlated with increased vascular permeability measurements of the lumens after sepsis plasma incubation. Permeability of EC incubated with media and with healthy plasma was comparable and exhibited high reproducibility, with a narrow range of permeability changes after incubation (media median [AUC] 349.5, IQR 27.9; healthy plasma median 363.1, IQR 14.9, Figure 1b–d). In contrast, EC incubated with plasma from either Sepsis+ARDS or Sepsis without ARDS patients exhibited significantly higher permeability (Sepsis+ARDS: median AUC 684.9, IQR 365.7; Sepsis without ARDS: median AUC 511.9, IQR 164.6) compared to healthy plasma (median AUC 363.1, IQR 14.9, *p* = 0.009 for difference with Sepsis+ARDS plasma, *p* = 0.02 for difference with Sepsis without ARDS plasma, Figure 1b–d). There was a wide range of permeability changes in response to sepsis plasma, reflecting heterogeneity in sepsis severity and host responses. Permeability of the Sepsis+ARDS group was not significantly higher (median 684.9, IQR 365.7, *p* > 0.9) than the Sepsis without ARDS group (median 511.9, IQR 164.6).

### 3.3 Endothelial activation and inflammatory cytokine analysis

**FIGURE 2:**
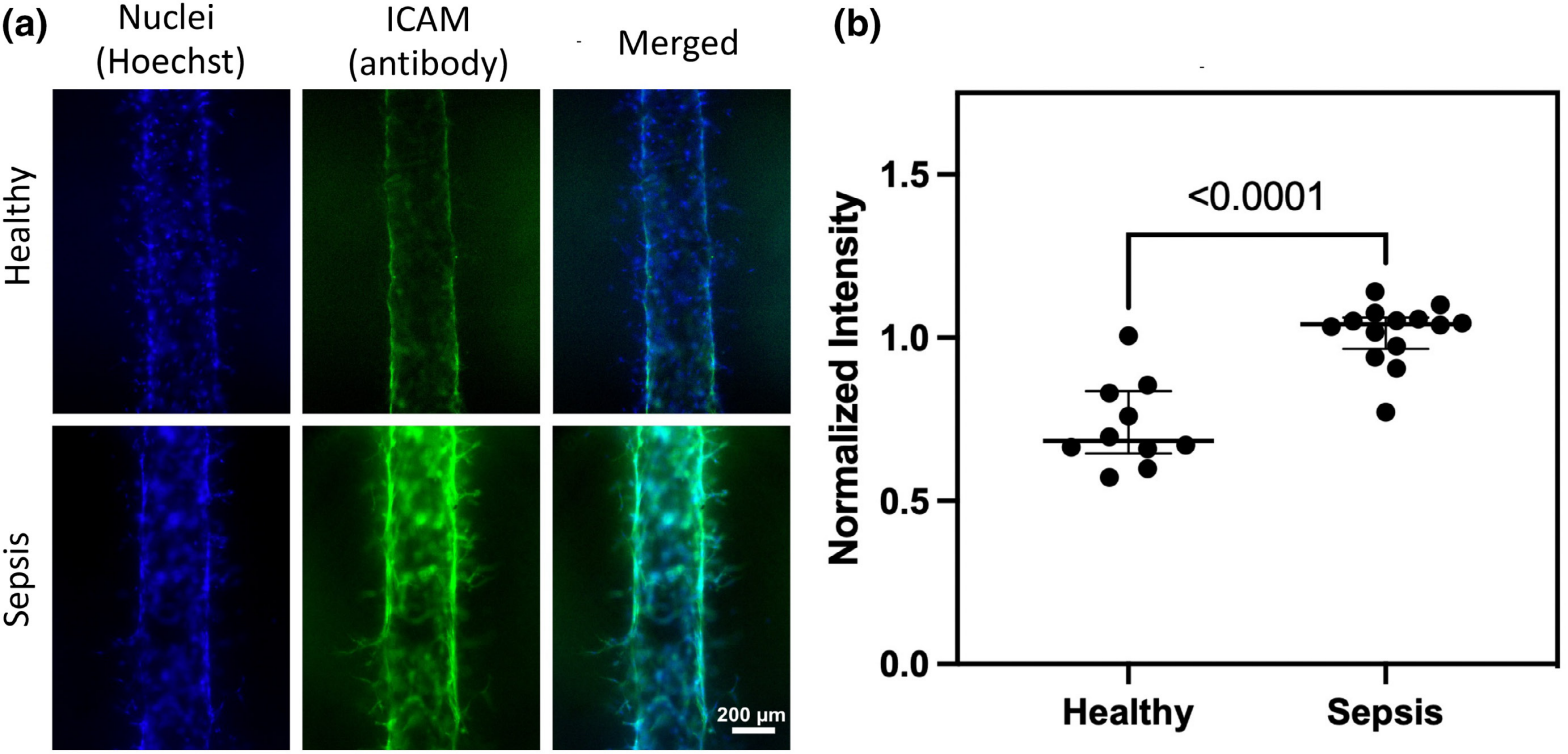
(a) ICAM staining on representative sepsis and healthy lumens. (b) Intensity of ICAM staining after incubation with representative healthy (*n* = 10 technical replicates) and sepsis plasma (*n* = 15 technical replicates) indicating significantly increased ICAM expression on sepsis lumens (MFI 0.7, IQR 0.15 vs. 1.04, IQR 0.07, *p* < 0.0001.)

**FIGURE 3:**
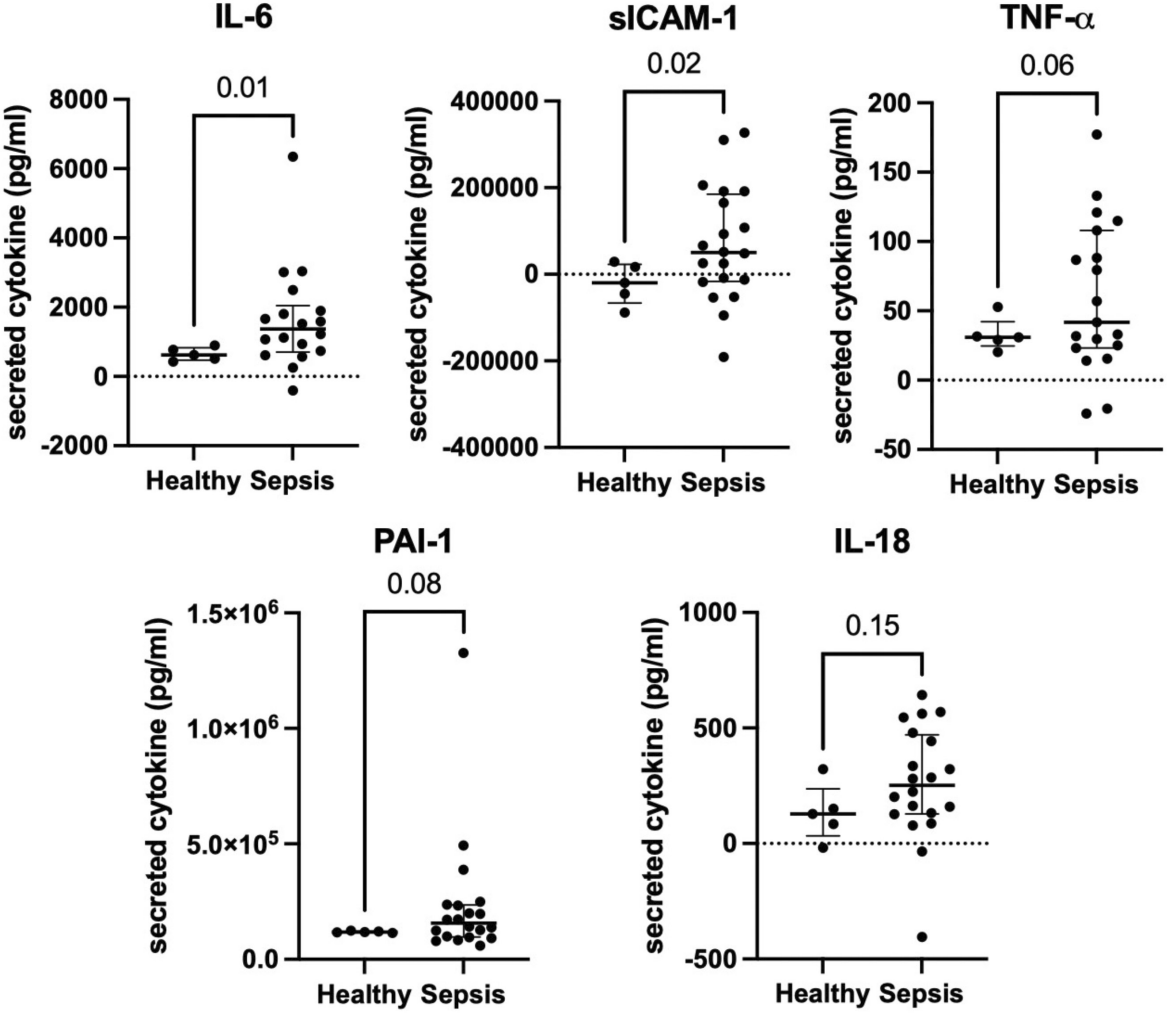
Sepsis plasma (*n* = 20) induces greater endothelial cell (EC) cytokine and endothelial inflammatory marker secretion compared with healthy plasma (*n* = 5). Levels were calculated as value obtained from the plasma after 16‐h incubation in the endothelial MPS minus the value obtained from the plasma prior to incubation in MPS. (IL‐6 sepsis median 1370.8 pg/mL, IQR 1088.4 vs. healthy 621.8 pg/mL, IQR 258.1, *p* = 0.01; sICAM‐1 sepsis median 50238.7 pg/mL, IQR 185539.3 vs. −19934.6 pg/mL, IQR 61657.0, *p* = 0.02; TNF‐*α* sepsis median 49.4 pg/mL, IQR 85.0 vs. healthy median 30.9 pg/mL, IQR 2.5, *p* = 0.06; PAI‐1 sepsis median 156488.4 pg/mL, IQR 135080.3 vs. healthy median 118532.9 pg/mL, IQR 3651.6, *p* = 0.08) Negative values indicate a decline in cytokine concentration from baseline to post‐incubation. Table S1 summarizes cytokine concentrations.

**FIGURE 4:**
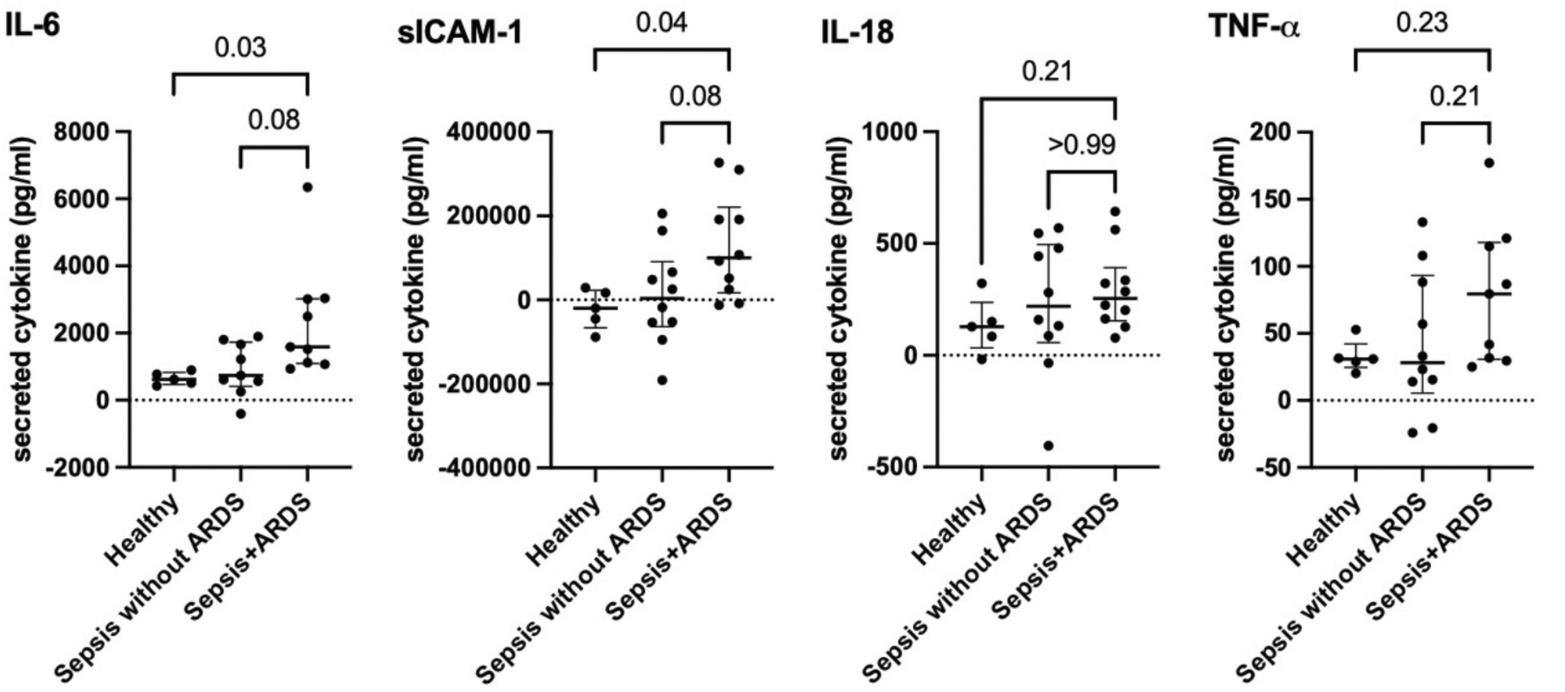
IL‐6 and sICAM‐1 concentrations were significantly increased in Sepsis+ARDS patient plasma (*n* = 10) compared with healthy plasma (*n* = 5) following incubation in the endothelial cell (EC) lumens (1584.2 pg/mL, IQR 1892.0 vs. 621.8 pg/mL, IQR 258.1, *p* = 0.03 and 100118.5 pg/mL, IQR 159832.3 vs. −19934.6 pg/mL, IQR 61657.0, *p* = 0.04 for IL‐6 and sICAM‐1 respectively). IL‐6 and sICAM‐1 concentrations were numerically higher in Sepsis + ARDS plasma than Sepsis without ARDS plasma (*n* = 10) following incubation in the EC lumens but did not reach statistical significance. Table S1 summarizes cytokine concentrations.

We next evaluated endothelial inflammatory responses to sepsis plasma. We stained ECs after incubation with healthy and sepsis plasma to determine ICAM‐1 expression. ICAM‐1 expression by ECs was increased significantly following incubation with sepsis plasma compared with healthy plasma (median 0.7 MFI, IQR 0.1 vs. 1.0, IQR 0.07, *p* < 0.0001, Figure 2a,b). We then quantified secretion of cytokines associated with inflammation and endothelial activation at baseline and after incubation in the EC lumens. The change from baseline to post‐incubation IL‐6 and sICAM‐1 concentrations was significantly higher in sepsis plasma compared with healthy plasma (median 1370.8 pg/mL, IQR 1088.4 vs. 621.8 pg/mL, IQR 258.1, *p* = 0.01 and 50238.7 pg/mL, IQR 185539.3 vs. −19934.6 pg/mL, IQR 61657.0, *p* = 0.02 for IL‐6 and sICAM‐1, respectively; Figure 3). TNF‐*α* and PAI‐1 concentrations were numerically higher in sepsis plasma but did not reach statistical significance. When stratified by presence or absence of ARDS within 5 days of ICU admission, the concentration of IL‐6 and sICAM‐1 concentrations were significantly increased in Sepsis+ARDS patient plasma compared with healthy plasma patient following incubation in the EC lumens (median 1584.2 pg/mL, IQR 1892.0 vs. 621.8 pg/mL, IQR 258.1, *p* = 0.03 and 100118.5 pg/mL, IQR 159832.3 vs. −19934.6 pg/mL, IQR 61657.0, *p* = 0.04 for IL‐6 and sICAM‐1, respectively; Figure 4). IL‐6 and sICAM‐1 concentrations were numerically higher in Sepsis+ARDS plasma than Sepsis without ARDS patient plasma following incubation in the EC lumens, but this difference did not reach statistical significance (Figure 4).

### 3.4 Correlation of permeability and inflammatory cytokine and endothelial activation markers

To determine the concordance of barrier function and endothelial activation, permeability and inflammatory cytokine and endothelial activation marker values for each were correlated. IL‐6 was moderately correlated with permeability (*R* ^2^ = 0.48, *p* = 0.006, Figure S2). sVCAM‐1 and TNF‐*α* was weakly correlated with permeability (*R* ^2^ = 0.38, *p* = 0.03; *R* ^2^ = 0.29, *p* = 0.04, respectively, Figure S2).

## 4 DISCUSSION

We present a physiologically plausible model of sepsis‐associated ARDS through use of a three‐dimensional lung endothelial MPS in combination with plasma from sepsis patients. Lung endothelial dysfunction plays a crucial role in the development of ARDS triggered by circulating factors released during sepsis and other systemic inflammatory conditions. Increasing evidence suggests that endothelial dysfunction can be effectively targeted to improve outcomes in sepsis and ARDS patients (Calfee et al., 2018; Sinha et al., 2023). However, while certain risk factors, such as extrapulmonary sepsis source or hyperinflammatory ARDS subphenotype, may be associated with increased endothelial dysfunction, it is difficult to identify clinically. Furthermore, understanding of mechanisms of endothelial dysfunction in ARDS are hampered by the inaccessibility of endothelial tissue. These factors limit identification of new candidate biomarkers to predict development of endothelial dysfunction and enrich for these patients in clinical trials of targeted therapies.

We aimed to overcome the challenges of studying human lung endothelial responses to sepsis through use of our high‐fidelity MPS to measure specific EC responses to sepsis patient plasma. Use of sepsis patient plasma, which has not been previously reported in a lung endothelial MPS, applies a physiological stimulus to endothelial dysfunction and reproduces the inherent variability and complexity of human sepsis responses. We utilized plasma collected immediately prior to or after ICU admission to assess early endothelial responses to sepsis, potentially preceding or coinciding with the development of lung injury. This may facilitate identification of driving mechanisms and predictive biomarkers of endothelial dysfunction. We demonstrated morphologic, functional, and secreted alterations in ECs after incubation with sepsis plasma, replicating clinically observed phenomena such as endothelial barrier dysfunction and upregulation of adhesion molecules. Additionally, we found significant increases in cytokines linked to the activation of specific inflammatory pathways in sepsis plasma compared to healthy plasma. Furthermore, differences in these cytokine levels and adhesion molecule levels were more pronounced between sepsis patients with and without ARDS. Increases in ICAM‐1 expression are consistent with the induction of endothelial activation, while higher concentrations of IL‐6 and PAI‐1 resemble molecular profiles demonstrated in human studies of extrapulmonary ARDS (Calfee et al., 2015). Our findings suggest a possible inflammatory phenotype of plasma from a subset of sepsis patients that may confer increased ARDS risk.

There are several important strengths of our study. Our use of plasma from sepsis patients with and without ARDS is unique in microphysiological models. The early sample collection occurred at the most proximal possible clinical timepoint offering the best opportunity to capture mechanisms that may drive organ injury and intervene before such injury occurs. The MPS offers several key advantages, including three‐dimensional structure that better replicates in vivo vascular architecture, ability to test multiple technical replicate lumens for reproducibility, and multiple physiologically relevant endpoints such as barrier dysfunction and adhesion molecule upregulation. Our use of human lung microvascular ECs is more clinically relevant to sepsis and ARDS pathophysiology than other commonly used EC lines, which have commonly included HUVECs and other non‐microvascular cell types. While other research groups have employed microfluidic models or MPS to study sepsis, most microfluidic assays targeting sepsis have concentrated on diagnostics rather than delving into the underlying biological mechanisms (Zhou et al., 2021). Additionally, some organotypic models have been used to investigate sepsis biology but have important differences with our study. In a study where TNF‐*α*‐treated brain microvascular ECs were used in a lumen model to mimic sepsis responses, they found decreased barrier function. However, this model relied on chemokine activation rather than using plasma from patients with sepsis (Pediaditakis et al., 2022). A recently published study that highlights the need for MPS to study ARDS also relied on non‐physiological stimuli to induce a generic inflammation response and lacks vasculature geometry (van Os et al., 2023). Additional studies have also focused on immune cell responses to septic inflammation (Kilpatrick & Kiani, 2020; Yang et al., 2024). However, lung microvascular EC responses to plasma in sepsis remain relatively unexplored. Finally, utilization of plasma from sepsis patients in our model encompasses a spectrum of infectious pathogens, varying disease severity, and heterogenous immune responses inherent to sepsis pathophysiology. These complexities are difficult to replicate in animal models.

There are several limitations of our study. In this proof‐of‐concept study, our sample size was small and lacked racial diversity. Additionally, health information regarding the healthy blood donors was not available. However, if unrecognized medical conditions such as diabetes, hypertension, and cardiovascular disease associated with increased plasma inflammatory mediators were present in the healthy donors, this would increase endothelial dysfunction and bias our results toward the null. Currently, our model operates in a static manner, but adding flow could better simulate physiological conditions. This could be accomplished in the future either by modifying the MPS to induce gravity‐driven flow or by incorporating a peristaltic pump. Use of flow creates shear stress that decreases endothelial permeability, although concurrent application of physiologic transvascular flow returns permeability to levels observed from static models (Akbari et al., 2018). Flow has also been shown to affect neutrophil adhesion in an MPS using sepsis patient neutrophils with buffer or cytomix stimulation (Yang et al., 2024); however, we chose to focus our model specifically on endothelial responses to plasma. While the MPS utilized is a reductive model that does not include immune cell‐mediated lung injury, we believe this represents a strength by isolating endothelial‐specific responses. Our system has the capacity to add in patient‐specific or donor leukocytes, a planned future direction.

In conclusion, our system offers significant insight into endothelial responses to sepsis and may facilitate identification of therapeutic targets and predictive biomarkers to anticipate organ dysfunction such as ARDS. We characterized cell morphology, relevant physiologic functional measures such as vascular permeability, and adhesion marker expression using minimal volumes of patient‐obtained plasma to construct a reductive but useful model of endothelial dysfunction. The inflammatory cytokine and endothelial activation markers measured in the sepsis plasma are consistent with pathophysiological profiles identified in extrapulmonary ARDS patient cohorts (Calfee et al., 2015), emphasizing the external validity and accuracy of our model. By replicating these processes in an accessible humanized in vitro model, we can directly assess causality of specific candidate mechanisms, identify and test potential therapeutic targets for endothelial dysfunction. The lung endothelial MPS may accelerate these critical steps to develop endothelial dysfunction into a treatable trait and improve care for critically ill sepsis and ARDS patients.

## FUNDING INFORMATION

Dr. Faust is funded by KL2TR002374 and UL1TR002373 awarded to UW ICTR through NIH NCATS.

## CONFLICT OF INTEREST STATEMENT

David J. Beebe holds equity in Bellbrook Labs LLC, Tasso Inc., Salus Discovery LLC, Lynx Biosciences Inc., Stacks to the Future LLC, Flambeau Diagnostics LLC, and Onexio Biosystems LLC.

## ETHICS STATEMENT

This study was reviewed and approved by the University of Wisconsin Institutional Review Board. Informed consent was obtained from all patients or their legally authorized representatives for collection of plasma and data collection.

## Supporting information


Figure S1.

Table S1.

Video S1.

Video S2.

## Data Availability

Source data for this study are not publicly available due to privacy restrictions. The source data are available to verified researchers upon request by contacting the corresponding author.
