# Supplementary material for: Modeling lung endothelial dysfunction in sepsis‐associated ARDS using a microphysiological system
Source: Physiol Rep. 2024 Jul 9;12(13):e16134. doi: 10.14814/phy2.16134 (PMC11233195; doi:10.14814/phy2.16134)
Supplement: Supplementary file 1 — Figure S1. [file PHY2-12-e16134-s003.pdf]

**Supplemental Figure 1**: Lung Endothelial MPS

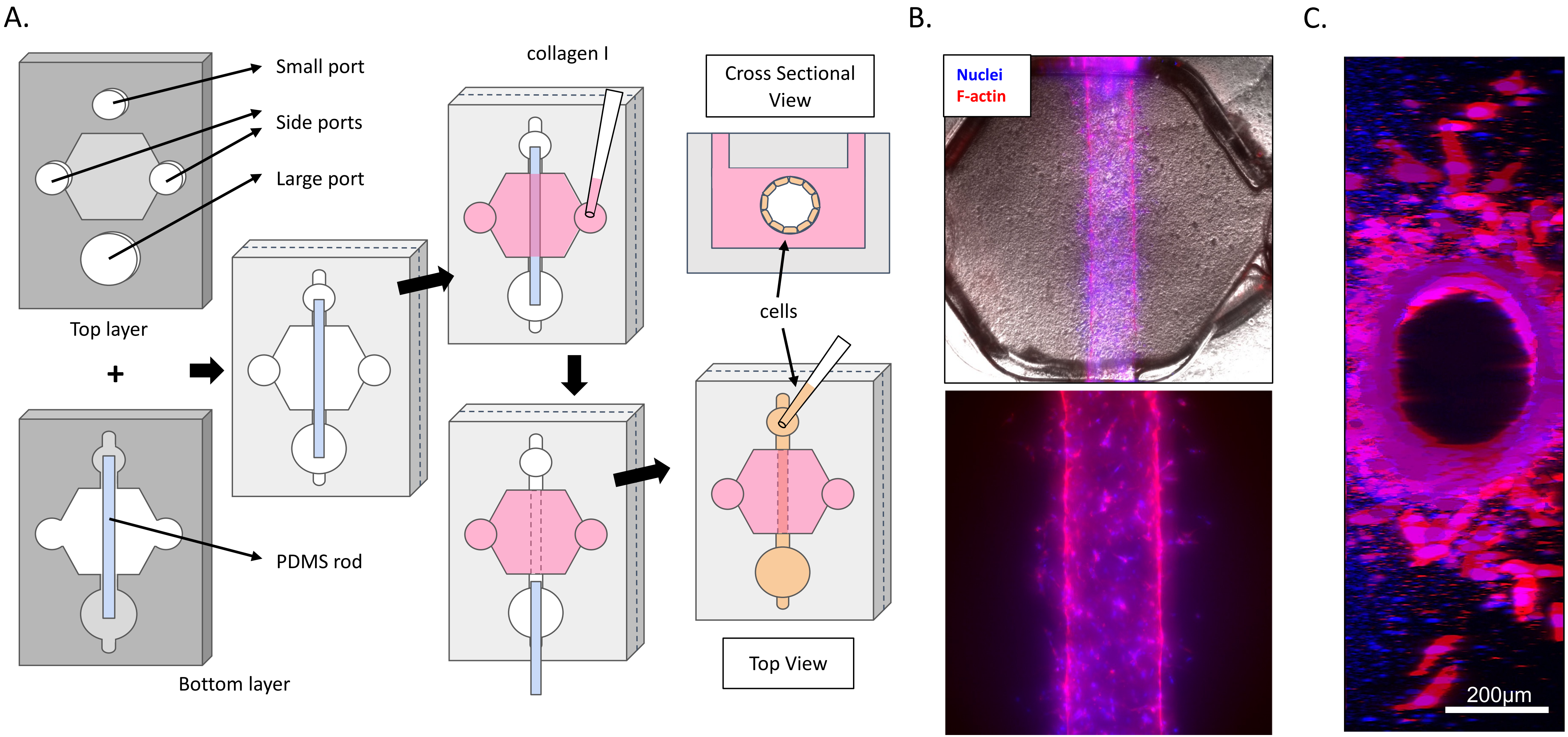


Supplemental Figure 1. A) Schematic of lung endothelial MPS. B) Images demonstrating coverage with endothelial cells prior to incubation of plasma in the lumens. C) Confocal microscopy of lung endothelial MPS lumen in cross section.

**Supplemental Figure 2:** Cytokine concentrations in plasma before (left) and after (right) incubation with EC

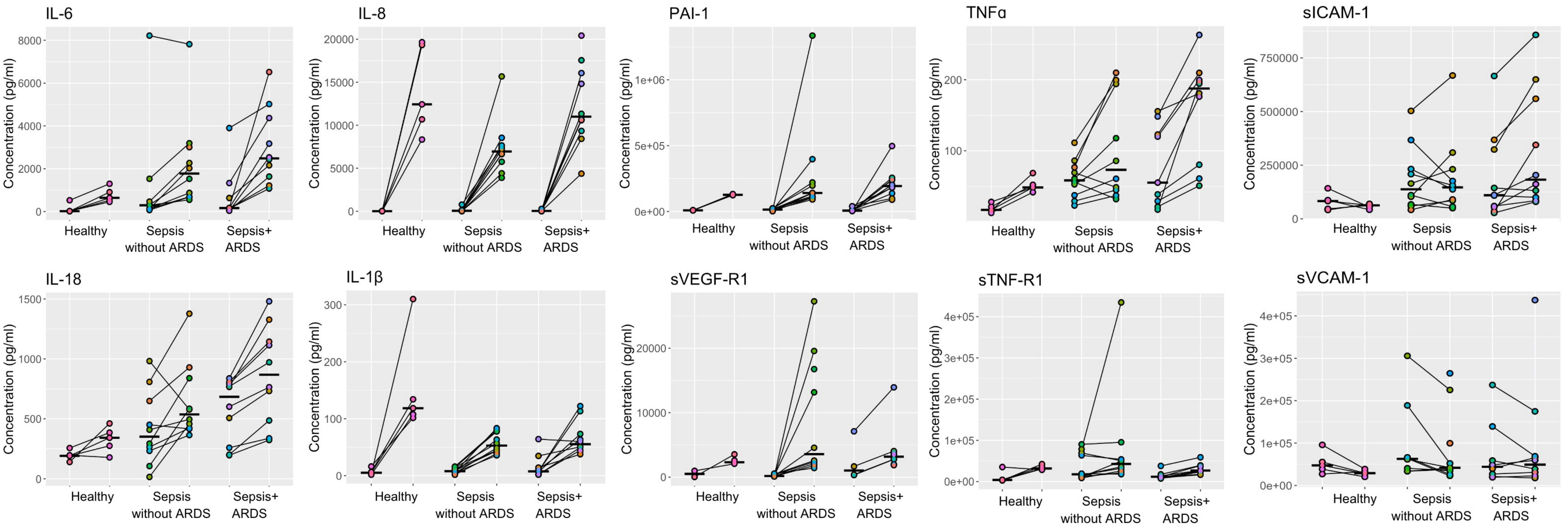

**Supplemental Figure 3:** Correlation of vascular permeability and endothelial activation markers

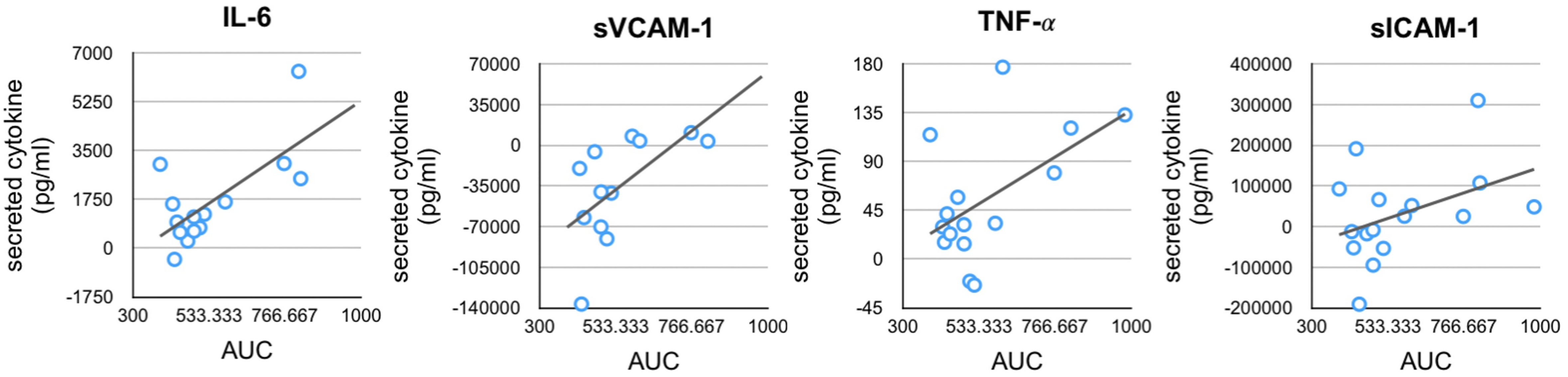


$R^2 = 0.48$
P = 0.006

$R^2 = 0.38$
P = 0.03

$R^2 = 0.29$
P = 0.04

$R^2 = 0.11$
P = 0.11
