# Supplementary figures and images for: Modeling lung endothelial dysfunction in sepsis‐associated ARDS using a microphysiological system
Source: Physiol Rep. 2024 Jul 9;12(13):e16134. doi: 10.14814/phy2.16134 (PMC11233195; doi:10.14814/phy2.16134)

Supplemental Table 1. Patient Characteristics


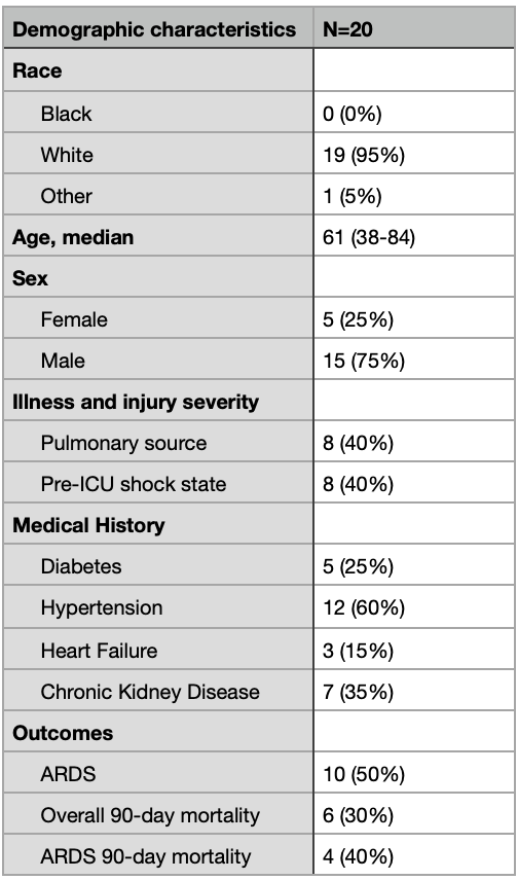

Supplement: Supplementary file 2 — Table S1. [file PHY2-12-e16134-s004.docx]
